# How birth outcomes among a cohort of Guatemalan women with a history of prior cesarean vary by mode or birth across different interpregnancy intervals

**DOI:** 10.1186/s12978-021-01153-4

**Published:** 2021-05-21

**Authors:** Margo S. Harrison, Ana Garces, Lester Figueroa, Jamie Westcott, Michael Hambidge, Nancy F. Krebs

**Affiliations:** 1 University of Colorado Anschutz Medical Campus, Aurora, CO USA; 2 Institute of Nutrition of Central America and Panama, Guatemala City, Guatemala; 3 University of Colorado Anschutz Medical Campus, Mail Stop B198-2, Academic Office 1, 12631 E. 17th Avenue, Rm 4211, Aurora, CO 80045 USA

**Keywords:** Mode of delivery after cesarean, Interpregnancy interval, Guatemala

## Abstract

**Objectives:**

Our objectives were to analyze how pregnancy outcomes varied by cesarean birth as compared to vaginal birth across varying interpregnancy intervals (IPI) and determine if IPI modified mode of birth.

**Methods:**

This secondary analysis used data from a prospective registry of home and hospital births in Chimaltenango, Guatemala from January 2017 through April 2020, through the Global Network for Women’s and Children’s Health Research. Bivariate comparisons and multivariable logistic regression were used to answer our study question, and the data was analyzed with STATA software v.15.1.

**Results:**

Of 26,465 Guatemalan women enrolled in the registry, 2794 (10.6%) had a history of prior cesarean. 560 (20.1%) women delivered by vaginal birth after cesarean with the remaining 2,233 (79.9%) delivered by repeat cesarean. Repeat cesarean reduced the risk of needing a dilation and curettage compared to vaginal birth after cesarean, but this association did not vary by IPI, all p-values > p = 0.05. Repeat cesarean delivery, as compared to vaginal birth after cesarean, significantly reduced the likelihood a woman breastfeeding within one hour of birth (AOR ranged from 0.009 to 0.10), but IPI was not associated with the outcome. Regarding stillbirth, repeat cesarean birth reduced the likelihood of stillbirth as compared to vaginal birth (AOR 0.2), but again IPI was not associated with the outcome.

**Conclusion:**

Outcomes by mode of delivery among a Guatemalan cohort of women with a history of prior cesarean birth do not vary by IPI.

## Plain English Summary

During a qualitative study where providers at one public hospital in Guatemala were interviewed about their beliefs, attitudes, and general practices regarding mode of delivery among women with a history of prior cesarean birth, providers reported that women with a short interval pregnancy (less than 18 or 24 months depending on provider interviewed) were not candidates for trial of labor after cesarean in their facility due to risk of adverse outcomes. We wished to test the hypothesis that adverse pregnancy outcomes are no more likely with an interval of shorter than 18 or 24 months compared to a longer interval, regardless of whether a woman delivers by vaginal or cesarean birth. This analysis suggests that outcomes can be worse for infants with vaginal birth after cesarean as compared to elective repeat cesarean birth, but these outcomes do not vary by interpregnancy interval. Therefore, our hypothesis was correct, and we look forward to disseminating this information to providers in the region in effort to improve evidence-based obstetric care.

## Introduction

During a qualitative study where providers at one public hospital in Guatemala were interviewed about their beliefs, attitudes, and general practices regarding mode of delivery among women with a history of prior cesarean birth, providers reported that women with a short interval pregnancy (less than 18 or 24 months depending on the provider interviewed) *were not candidates* for trial of labor after cesarean in their facility (data under review, Harrison). Providers explained to us that they were deciding about mode of birth among women with a prior cesarean based on IPI alone, which is why we conducted this analysis.

Regarding what the literature says about mode of birth and IPI, three prior studies have shown a risk of uterine rupture associated with pregnancy spacing among women with a history of prior cesarean delivery; the first reports an increased risk of rupture with an interdelivery interval less than 16 months; the second found an interval of less than 19 months was associated with a 2.25% risk of uterine rupture compared to a 1.05% risk with an interval longer than 19 months; and there was an increased odds of rupture at less than 18 months (AOR 3.0, CI 1.3–7.2) compared to greater than 18 months (AOR 1.1, CI 0.4–3.2) in the final analysis [1–3]. Guidance from the American College of Obstetricians and Gynecologists (ACOG) discusses interpregnancy interval (IPI) in relation to likelihood of vaginal birth after cesarean success, but not related to pregnancy outcomes such as uterine rupture. The World Health Organization recommends 24 months between a livebirth and subsequent conception but does not give specific recommendations in the setting of a prior cesarean birth [4].

Given the current common practice at this facility in Guatemala was not to offer trial of labor after cesarean if a woman delivered a baby in the past 18 or 24 months and lack of clear guidance on this topic, the aim of this analysis was to observe mode of delivery and associated maternal and perinatal/neonatal outcomes by IPI among women with a history of prior cesarean delivery in a large Guatemalan cohort. Our objectives were to analyze: (1) how IPI was associated with mode of delivery, and (2) how maternal and perinatal/neonatal outcomes varied by mode of delivery and IPI. We hypothesized (based on prior research) that repeat cesarean delivery would be associated with better neonatal outcomes than vaginal delivery after cesarean, but that these findings would not vary by IPI. Another way to say this is do birth outcomes varies by mode of delivery stratification by interpregnancy interval, and is there an interaction between the covariates.

## Methods

### Study design

This analysis was conducted using data from a prospective study conducted in communities in Chimaltenango, Guatemala from January 2017 through April 2020, through the Global Network for Women’s and Children’s Health Research, Maternal and Newborn Health Registry (MNHR) [5].

### Setting

The Global Network’s prospective registry, the MNHR, includes pregnancy related data and outcomes from rural or semi-urban geographical areas. The Guatemalan site includes 17 distinct clusters served by one referral hospital, 30 health centers, and 42 health posts [5]. Each community generally represents the catchment area of a primary healthcare center, and about 300 to 500 annual deliveries [5]. The objective of the MNHR is to enroll pregnant women as early as possible during the pregnancy and to obtain data on pregnancy outcomes for all deliveries of registered women, regardless of delivery location (i.e., home, health clinic, or hospital) [5].

### Population

Only women with a history of prior cesarean delivery with information on date of last delivery and mode of delivery of the enrollment pregnancy were included in this analysis. If date of last delivery or mode of delivery data was missing, women were excluded from the analysis.

### Recruitment

The population studied included women screened for the MNHR who were eligible, consented, and delivered in the study period [5]. Data were excluded from women who were enrolled but lost to follow-up prior to delivery, maternal deaths prior to labor and delivery, miscarriages, medically terminated pregnancies, and those with missing data for delivery mode or date of last delivery. Only women with a history of prior cesarean birth were included in the analysis per the study question.

### Primary outcomes

The primary outcome of this analysis was mode of delivery among women with a history of prior cesarean. We wish to observe what characteristics predicted repeat cesarean birth. IPI was an independent variable and mode birth was the dependent variable in this initial analysis.

### Secondary outcomes

The secondary outcomes were maternal and perinatal/neonatal outcomes (the dependent variable) that resulted after mode of birth, which now because an independent variable in our secondary modeling. Maternal outcomes included uterotonic use, blood transfusion, dilation and curettage, magnesium administration, hysterectomy, severe infection, postpartum infection, seizure, unplanned hospitalization, and death by 42 days postpartum; neonatal outcomes included: fetal status at birth, bag and mask resuscitation, breastfeeding within an hour of birth, neonatal antibiotic administration, CPAP and/or oxygen administration, ventilation, and death by 42 days of life. We wished to observe how the risk of those outcomes changed by varying IPIs, so we present individual models where IPI is included first as a continuous variable, and subsequently as a dichotomous variable. We tested the IPI as less than compared to greater than 12 months, followed by comparisons at 18 and 24 months to observe whether this change in the independent variable definition was associated with differences in maternal and perinatal/neonatal outcomes. We also tested the interaction between IPI and mode of birth as well.

### Analysis plan

We used descriptive statistics to produce counts and percentages regarding mode of delivery among women with a history of prior cesarean birth in the registry. Then we observed independent variables associated with mode of delivery, and performed bivariate comparisons of sociodemographic and antenatal covariates, and intrapartum characteristics that we hypothesized might be associated with mode of birth. P-values were obtained from bivariate comparisons as a function of each individual risk factor using Kruskal–Wallis, Fisher’s Exact, or Chi-squared tests depending on variable type.

All risk factors that occurred before delivery and might be associated with mode of delivery were including in a logistic regression associated with repeat cesarean delivery (p < 0.05 from the individual risk factor bivariate comparisons). We performed this first considering IPI as a continuous variable and second as a dichotomous variable comparing an interval of < 12 months vs ≥ 12 months, a dichotomous variable comparing an interval of < 18 months vs ≥ 18 months, and a dichotomous variable comparing an interval of < 24 months vs ≥ 24 months.

We then used individual backwards stepwise logistic regressions to observe the association of mode of delivery with maternal and perinatal/neonatal outcomes that were significantly different in bivariate comparisons by mode of delivery (p < 0.05 from the individual risk factor bivariate comparisons). Each of these outcomes was first considered using IPI as a continuous variable and second as a dichotomous variable comparing an interval of < 12 months vs ≥ 12 months, a dichotomous variable comparing an interval of < 18 months vs ≥ 18 months, and a dichotomous variable comparing an interval of < 24 months vs ≥ 24 months. We also included an interaction term in the model between IPI and mode of birth to observe for effect modification. No methods were used to adjust for any potential bias. All data analyses were performed with STATA software v.15.1. (STATA Corp, College Station, TX, USA).

## Results

**Fig. 1:**
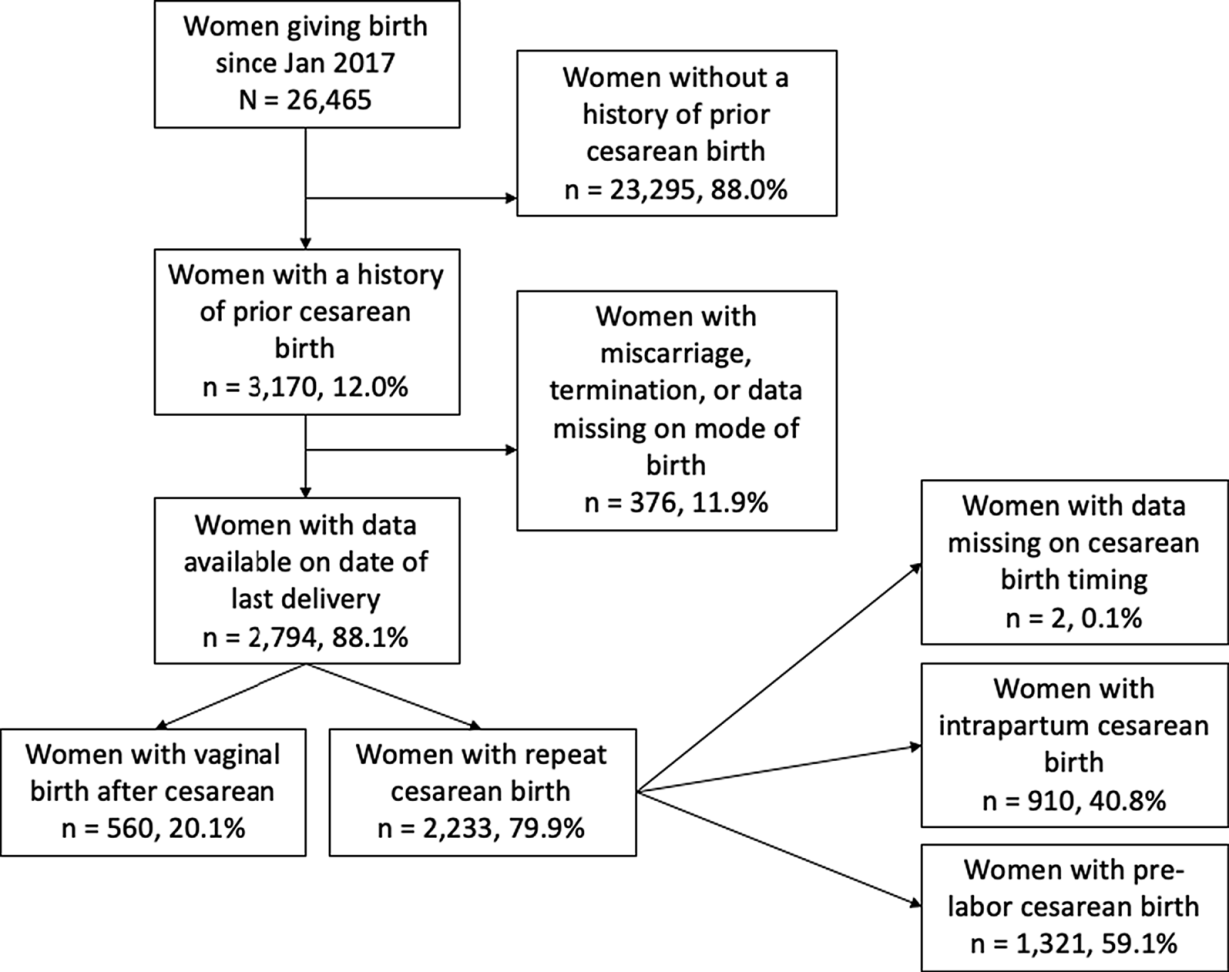
Population of women with a history of prior cesarean birth and mode of subsequent delivery at the Guatemalan site of the Global Network for Women’s and Children’s Health Research, Maternal and Newborn Health Registry

We present a flow diagram of the population of women included in this study in Fig. 1. Between January 2017 and April 2020, 26,465 women delivered in the Guatemalan clusters of the MNHR. 3170 women, which is 12.0% of the MNHR population, had a history of prior cesarean birth. Of these women, 2794 (88.1%) had data available both on mode of birth of the index pregnancy as well as the date of their prior birth, which we used to calculate IPI. About a fifth of these women (560, 20.1%) delivered by vaginal birth after cesarean with the remaining 2233 (79.9%) delivering by repeat cesarean delivery.

**Fig. 2:**
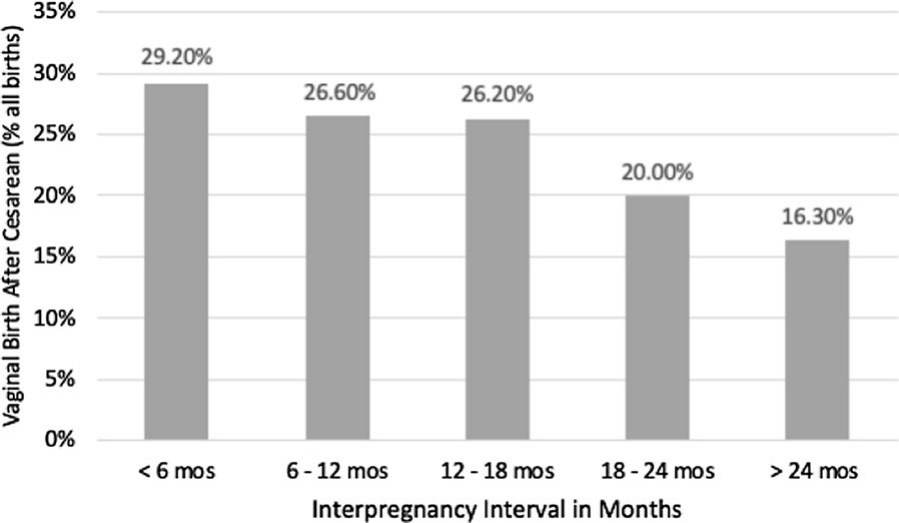
Proportion of vaginal birth after cesarean by 6-month IPIs with trend test. Univariate logistic regression of time interval as categorical independent variable on mode of delivery: 20% increase in odds of repeat cesarean birth per six-month interval (UOR 1.2, CI [1.2, 1.3], p < 0.001)

We then illustrate mode of birth among women with a history of prior cesarean delivery as a function of IPI (time since a woman’s last delivery) in Fig. 2. A univariate logistic regression of IPI (categorical variable of 6-month intervals) on mode of delivery found that with each successive 6-month interval, repeat cesarean delivery became 20% more likely (UOR 1.2, p < 0.001).

Sociodemographic and obstetric/labor characteristics of the population overall and by mode of delivery are presented in Table 1. The population was overall median age 27 with interquartile range (IQR) 23 to 31 years. Most women had some schooling (93.5%), just over half were primiparous (52.7%), and almost two-thirds women (73.1%) were of normal or overweight body mass index (BMI). Women who delivered by repeat cesarean delivery (as compared to vaginal delivery after cesarean) were statistically more likely to be younger (median age 27 versus 28), to have had schooling (95.2% versus 86.8%), to be less parous (parity 3 + 11.6% versus 44.8%), and more likely to be overweight or obese (71.0% versus 61.2%), p < 0.001.

**Table 1:** Population characteristics of women with a history of prior cesarean birth overall and by mode of delivery, January 2017–April 2020

	Total women with history of cesarean N = 2793	Vaginal birth after cesarean n = 560, 20.1%	Repeat cesarean birth n = 2233, 79.9%	p-value
Sociodemographics				
Age in years [IQR]	27 [23,31]	28 [23,33]	27 [23,31]	< 0.001^a^
Schooling n, %				< 0.001^c^
Illiterate	157, 5.6%	63, 11.2%	94, 4.2%	
Literate, no school	24, 0.9%	11, 2.0%	13, 0.6%	
Schooling	2612, 93.5%	486, 86.8%	2126, 95.2%	
Parity n, %				< 0.001^c^
1	1472, 52.7%	161, 28.8%	1311, 58.7%	
2	812, 29.1%	148, 26.4%	664, 29.7%	
3 +	509, 18.2%	251, 44.8%	258, 11.6%	
BMI kg/m^2^				< 0.001^b^
< 18.5	10, 0.4%	0, 0.0%	10, 0.4%	
18.5–24.9	856, 30.6%	217, 38.8%	639, 0.6%	
25–29.9	1188, 42.5%	222, 39.6%	966, 43.3%	
≥ 30	739, 26.5%	121, 21.6%	618, 27.7%	
Obstetric characteristics				
IPI in months [IQR]	27.3 [15.2,46.3]	21.7 [12.9,39.2]	29.1 [16.2,48.1]	< 0.001^a^
Antenatal Care n, %	2708, 97.0%	512, 91.4%	2196, 98.3%	< 0.001^c^
Multiple Gestation n, %	27, 1.0%	4, 0.7%	23, 1.0%	0.63^b^
Female sex of baby n, %	1386, 49.6%	285, 50.9%	1101, 49.3%	0.10^c^
Missing	3, 0.1%	2,0.4%	1, 0.1%	
Birthweight in grams [IQR]	2870 [2610,3120]	2855 [2585,3105]	2890 [2610,3120]	0.21^a^
Missing	7, 0.3%	5, 0.9%	2, 0.1%	
Term Gestational Age n, %	2609, 93.4%	518, 92.5%	2091, 93.6%	0.33^c^
Obstructed Labor n, %	114, 4.1%	13, 2.3%	101, 4.5%	0.02^c^
Antepartum Hemorrhage n, %	6, 0.2%	1, 0.2%	5, 0.2%	1.0^b^
Hypertensive Disease n, %	164, 5.9%	6, 1.1%	158, 7.1%	< 0.001^c^
Induction of Labor n, %	44, 1.6%	22, 3.9%	22, 1.0%	< 0.001^c^
Referred in Labor n, %				< 0.001^c^
Yes	485, 17.4%	56, 10.0%	429, 19.2%	
Missing	1, 0.1%	0, 0.0%	1, 0.1%	
Attendant n, %				< 0.001^b^
Family	1, 0.05%	1, 0.2%	0, 0.0%	
Non-OB MD	120, 4.3%	52, 9.3%	68, 3.1%	
Nurse/Nurse Midwife	4, 0.1%	4, 0.7%	0, 0.0%	
OB	2318, 83.0%	153, 27.3%	2165, 96.9%	
Other	3, 0.1%	3, 0.5%	0, 0.0%	
Self	1, 0.05%	1, 0.2%	0, 0.0%	
Traditional birth attendant	346, 12.4%	346, 61.8%	0, 0.0%	
Delivery location n, %				< 0.001^b^
Clinic/Health Center	2, 0.1%	1, 0.2%	1, 0.1%	
Home	351, 12.6%	351, 62.7%	0, 0.0%	
Hospital	2272, 81.3%	195, 34.8%	2077, 93.0%	
Other	168, 6.0%	13, 2.3%	155, 6.9%	

*Note* All tests performed excluding missing data

^a^Kruskall-Wallis

^b^Fisher’s exact

^c^chi^2^

Regarding obstetric and labor characteristics (Table 1) overall women had an IPI of 27 months (IQR 15 to 46 months), most had antenatal care (97.0%) and singleton gestations (99.0%), birthweight of their babies was median 2870 g (IQR 2610 to 3120 g) of mostly term infants (93.4%). Only 1.6% of the population was induced and a majority were delivered by obstetricians (83.0%) in the hospital setting (81.3%). When comparing women who delivered by repeat cesarean delivery compared to vaginal delivery after cesarean, they differed significantly on IPI (29 months versus almost 22 months), antenatal care (98.3% versus 91.4%), women experiencing obstructed labor (4.5% versus 2.3%) and induction of labor (1.0% versus 3.9%), hypertensive disease (7.1% versus 1.1%), and referral in labor (19.2% versus 10.0%), p < 0.05. Women delivered by repeat cesarean delivery were more likely to be delivered by an obstetrician (96.9% versus 27.3%) and in the hospital (93.0% versus 34.8%), p < 0.001.

Multivariable modeling of repeat cesarean birth including all variables occurring prior to delivery significant in bivariate comparisons (age, education, parity, BMI, prenatal care attendance, obstructed labor, hypertensive disease, induction of labor, and referred to the facility from another delivery setting) are shown in Table 2. The table shows the results of variables significant in the multivariable model as well as IPI, which was included in the model first as a continuous variable (column 1) and subsequently as a dichotomous variable defined by 12, 18, and 24 months. Increasing education, BMI, receipt of antenatal care, obstructed labor and hypertensive disease were associated with an increased odds of repeat cesarean delivery across all interpregnancy intervals, p < 0.05). Increasing parity and induction of labor were associated with a reduced risk of repeat cesarean, p < 0.05. Delivery provider and delivery location were dropped from the model as only physicians (compared to non-physicians) performed cesarean delivery and cesarean births only occur in an operating room in this region; including these covariates in the model prevented in from converging.

**Table 2:** Multivariable logistic regression of the association of risk factors significant in bivariate comparisons with repeat cesarean birth varying by IPI

	IPI as Continuous Variable		IPI as Dichotomous Variable (< 12 vs $\ge$ 12 months)		IPI as Dichotomous Variable (< 18 vs $\ge$ 18 months)		IPI as Dichotomous Variable (< 24 vs $\ge$ 24 months)	
	aOR	95% CI	aOR	95% CI	aOR	95% CI	aOR	95% CI
Predicting Repeat Cesarean Birth (ref: vaginal birth after cesarean)	1.0	1.0,1.0	1.5	1.1,1.9	1.5	1.2,1.8	1.3	1.1,1.7
Literacy and Schooling (ref: illiterate)	1.3	1.1,1.6	1.3	1.1,1.6	1.3	1.1,1.6	1.3	1.1,1.6
Increase in parity of 1 birth (continuous variable)	0.4	0.4,0.5	0.3	0.3,0.4	0.3	0.3,0.4	0.3	0.3,0.4
Increase in BMI of 1 category (ref: underweight)	1.4	1.2,1.8	1.4	1.2,1.7	1.4	1.2,1.6	1.4	1.2,1.6
Received any antenatal care (ref: no antenatal care)	4.2	2.5,6.9	4.2	2.5,6.9	4.0	2.4,6.6	4.1	2.5,6.7
Experienced obstructed labor (ref: no obstructed labor)	2.1	1.1,4.3	2.1	1.1,4.4	2.1	1.1,4.4	2.1	1.1,4.4
Induction of labor (ref: not induced)	0.1	0.1,0.2	0.2	0.1,0.3	0.2	0.1,0.3	0.1	0.1,0.3
Hypertensive disease (ref: no hypertensive disease)	3.5	1.3,9.5	4.1	1.7,10.1	4.2	1.7,10.3	4.2	1.7,10.3
Referred in labor (ref: not referred in labor)	1.9	1.3,2.8	1.9	1.3,2.8	1.9	1.3,2.8	1.9	1.3,2.8

*Note* All variables included in model that were significant in bivariate comparisons (Table 1) of mode of birth: age, education, parity, body mass index, prenatal care attendance, obstructed labor, hypertensive disease, induction of labor, referred to facility from another setting. Birth attendant and location of delivery were dropped as physician providers are the only ones providing cesarean birth in the hospital with zero cells for non-physician providers and non-facility cesareans

Bivariate comparisons of maternal and perinatal/neonatal outcomes by mode of delivery are shown in Table 3. In the overall population most maternal outcomes were rare (< 2.0%), but 5.2% of women in the cohort were treated with magnesium sulfate for seizure prophylaxis and many women were treated with uterotonics (86.5%). In bivariate comparisons, women varied by mode of delivery on uterotonic receipt (98.2% of repeat cesareans versus 40.0% of vaginal deliveries after cesarean) and dilation and curettage (0.1% of cesareans versus 3.2% of vaginal deliveries). With respect to neonatal outcomes, most babies were born alive (97.8%), 21.1% were breastfed within an hour of delivery, 4.1% of infants required neonatal antibiotics, and the remainder of adverse outcomes occurred rarely at less than a 2% prevalence. In bivariate comparisons, fetal status at delivery varied by mode of delivery (stillbirths in 1% of cesareans versus 3.9% of vaginal deliveries), as did breastfeeding within one hour of delivery (6.1% of cesareans versus 82.9% of vaginal deliveries), p < 0.001.

**Table 3:** Maternal and perinatal outcomes of women with a history of prior cesarean birth overall and by mode of delivery, January 2017–April 2020

	Total women with history of caesarean N = 2793	Vaginal birth after caesarean n = 560, 20.1%	Repeat cesarean birth n = 2233, 79.9%	p-value
Maternal outcomes				
Uterotonics n, %	2416, 86.5%	224, 40.0%	2192, 98.2%	< 0.001^b^
Blood transfusion n, %	29, 1.0%	4, 07%	25, 1.1%	0.49^b^
D&C/suction n, %	21, 0.8%	18, 3.2%	3, 0.1%	< 0.001^b^
Magnesium n, %	146, 5.2%	6, 1.1%	140, 6.3%	< 0.001^b^
Hysterectomy n, %	14, 0.5%	1, 0.2%	13, 0.6%	0.33^b^
Severe infection n, %	35, 1.3%	3, 0.5%	32, 1.4%	0.09^b^
Postpartum infection n, %	9, 0.3%	2, 0.4%	7, 0.3%	1.0^b^
Missing	83, 3.0%	15, 2.7%	68, 3.1%	
Seizure n, %	3, 0.1%	0, 0.0%	3, 0.1%	1.0^b^
Missing	83, 3.0%	15, 2.7%	68, 3.1%	
Unplanned hospitalization n, %	23, 0.8%	6, 1.1%	17, 0.8%	0.43^b^
Missing	83, 3.0%	15, 2.7%	68, 3.1%	
Death by 42 days n, %	1, 0.1%	1, 0.2%	0, 0.0%	0.20^b^
Missing	83, 3.0%	15, 2.7%	68, 3.1%	
Neonatal outcomes				
Fetal status n, %				< 0.001^b^
Born alive, alive	2732, 97.8%	526, 93.9%	2206, 98.8%	
Born alive, neonatal demise	17, 0.6%	12, 2.1%	5, 0.2%	
Stillbirth	44, 1.6%	22, 3.9%	22, 1.0%	
Bag and mask resuscitation n, %	31, 1.1%	9, 1.6%	22, 1.0%	0.21^b^
Missing	1, 0.1%	0, 0.0%	1, 0.1%	
Breastfeed within an hour n, %	581, 21.1%	446, 82.9%	135, 6.1%	< 0.001^c^
Neonatal antibiotics n, %	114, 4.1%	16, 2.9%	98, 4.4%	0.10^c^
CPAP n, %	7, 0.3%	1, 0.2%	6, 0.3%	1.0^b^
Missing	2, 0.1%	1, 0.2%	1, 0.1%	
Oxygen n, %	125, 4.5%	18, 3.2%	107, 4.8%	0.11^c^
Ventilation n, %	15, 0.5%	3, 0.5%	12, 05%	1.0^b^
Missing	1, 0.1%	0, 0.0%	1, 0.1%	
Death by 42 Days n, %	39, 1.4%	10, 1.8%	29, 1.3%	0.31^c^
Missing	143, 5.1%	48, 8.6%	95, 4.3%	

^a^Kruskall-Wallis

^b^Fisher’s exact

^c^chi^2^

*Note* All tests performed excluding missing data

Individual logistic regressions of the association of repeat cesarean delivery with the maternal outcomes of interest (those significant in bivariate comparisons) adjusted for covariates significant in bivariate comparisons are listed in Table 4. Maternal outcomes that varied by mode of delivery were uterotonic use, performance of dilation and curettage, and administration of magnesium sulfate. The likelihood of the outcomes by varying IPI definitions are shown. Each logistic regression was performed first with IPI as a continuous variable and then as a dichotomous variable (set at 12 months, 18 months, and 24 months). The likelihood of the outcomes did not vary with IPI definition, but IPI and mode of birth did interact when IPI was considered as a continuous variable (AORD 0.98 [0.98, 0.99]) suggesting that IPI modifies mode of birth in this context.

**Table 4:** Individual logistic regressions of maternal uterotonic administration, dilation and curettage, and magnesium sulfate in the setting of repeat cesarean birth by varying IPIs

	IPI as continuous variable		IPI as dichotomous variable (< 12 vs $\ge$ 12 months)		IPI as dichotomous variable (< 18 vs $\ge$ 18 months)		IPI as dichotomous variable (< 24 vs $\ge$ 24 months)	
	aOR	95% CI	aOR	95% CI	aOR	95% CI	aOR	95% CI
Odds of Needing Uterotonics (ref: no uterotonics)	1.0^a^	0.99,1.0	1.2	0.8,1.8	1.1	0.8,1.5	1.0	0.7,1.4
Odds of Needing D&C (ref: no D&C)	1.0	0.99,1.0	1.5	0.4,5.2	1.8	0.6,5.5	2.2	0.7,6.3
Odds of Needing MgSO_4_ (ref: no MgSO_4_)	1.0	0.99,1.0	0.3	0.1,2.8	2.5	0.8,7.3	2.0	0.7,5.6

*Note* Adjusted for education, parity, body mass index, prenatal care, obstructed labor, induction of labor, hypertensive disease, and referral in labor; birth attendant and location of delivery were dropped from the model as physician providers are the only ones providing cesarean birth in the hospital with zero cells for non-physician providers and non-facility cesareans

*D&C* dilation & curettage, *MgSO*_4_ magnesium sulfate

^a^Interpregnancy interval interacted with mode of birth in the continuous IPI model, suggesting that mode of birth was modified by interpregnancy interval, p = 0.04 (AOR 0.98 [0.98.0.99], p = 0.04)

The results of individual logistic regressions of the association of repeat cesarean delivery with the neonatal outcome of interest (those significant in bivariate comparisons) adjusted for IPI and covariates significant in bivariate comparisons are presented in Table 5. Perinatal outcomes that varied by mode of delivery were breastfeeding within one hour and fetal status at delivery. Each logistic regression was performed first with IPI as a continuous variable and then as a dichotomous variable (set at 12 months, 18 months, and 24 months). The likelihood of the outcomes did not vary with IPI definition. Regarding stillbirth, IPI was not associated with the outcome across all definitions, although it marginally (but not statistically) reduced stillbirth at intervals of greater than compared to less than 12 months (AOR 0.5 95% CI [0.2, 1.1], p = 0.08).

**Table 5:** Individual logistic regressions of breastfeeding and stillbirth in the setting of repeat cesarean birth by varying IPIs

	IPI as continuous variable		IPI as dichotomous variable (< 12 vs $\ge$ 12 months)		IPI as dichotomous variable (< 18 vs $\ge$ 18 months)		IPI as dichotomous variable (< 24 vs $\ge$ 24 months)	
	aOR	95% CI	aOR	95% CI	aOR	95% CI	aOR	95% CI
Odds of Breastfeeding by 1 Hour (ref: did not breastfeed)	1.0	1.0,1.0	1.4	1.0,2.1	1.0	0.8,1.4	1.1	0.8,1.5
Odds of Stillbirth (ref: no stillbirth)	1.0	0.98,1.0	0.5	0.2,1.1	0.8	0.4,1.5	0.8	0.4,1.6

*Note* Adjusted for education, parity, body mass index, prenatal care, obstructed labor, induction of labor, hypertensive disease, and referral in labor; birth attendant and location of delivery were dropped from the model as physician providers are the only ones providing cesarean birth in the hospital with zero cells for non-physician providers and non-facility cesareans

## Discussion

The main findings of this analysis were that there was an overall vaginal delivery after cesarean rate of 19.6% (that declined from 29.2% down to 16.3% over each successive six month interval between pregnancies, p < 0.001), and that repeat cesarean delivery compared to vaginal delivery after cesarean did not result in a difference in adverse pregnancy outcomes by varying IPIs. Therefore, based on outcomes available in the MNHR and this analysis, women should not be precluded from attempting trial of labor after cesarean by only considering IPI, alone.

Women with a longer interval in this analysis had 40% increased odds of repeat cesarean delivery, p = 0.03. It is interesting that per our qualitative research (which represents only one facility), women only become eligible for trial of labor after 18 or 24 months, depending on the provider. A study from the Netherlands found that an IPI of less than 24 months is not associated with a decreased success of vaginal delivery after cesarean, but success rates decrease when the interval increases, which is what we found in our analysis as well [6]. In a study of a population in California, USA, interdelivery intervals of less than 19 months were associated with a decreased rate of vaginal delivery after cesarean only in patients who underwent induction, but not for women in spontaneous labor [7]. While we did not address induction of labor in our analysis, we also found that rates of successful vaginal delivery after cesarean were higher in women with shorter interval pregnancies (in an unadjusted comparison, Fig. 2). We hypothesize that this finding is due to delivery location and spontaneity of labor and feel this is an area ripe for future research. For example, many of the women achieving successful VBAC delivered in the home setting, which is consistent with prior research [8]. We believe this may be an opportunity to improve outcomes of home VBAC by increasing the skill level of the traditional birth attendants, which has been associated with reduced morbidity and mortality [9].

The finding that vaginal delivery after cesarean compared to repeat cesarean delivery is associated with an increased need for dilation and curettage is not a novel or surprising finding. Retained placenta occurs in about 1–3% of vaginal deliveries compared to less than 1% of cesarean deliveries [10, 11]. What was important to note about this finding is the significance and odds of the complication did not vary by IPI. This finding suggests that ultrasound after vaginal delivery may be a clinical practice worth considering as part of a quality improvement program in this population [12, 13]. Our findings were similar regarding the odds of breastfeeding within one hour after delivery in that the outcome was not novel, and it did not vary by IPI. It is more common that women breastfeed earlier after a vaginal delivery than a cesarean, and again, if the population under study desires to address this issue, interventions targeted at this outcome exist for replication [14–17]. It is notable that at the 12-month timepoint, an interval longer than a year was marginally (not statistically) associated with an increased odds of breastfeeding after delivery.

While no outcomes varied by mode of delivery with respect to IPI, the only borderline result concerned stillbirth. Women with an interval of greater than 12 months between delivery and subsequent conception had a marginal statistically significant decrease in stillbirth. This suggests that if providers want to counsel women on trial of labor with respect to IPI, they would be justified in citing a trend toward an increase in stillbirth at less than 12 months, but a recommendation against trial of labor for this reason would not be supported by the results of our analysis. While a concerning finding that vaginal delivery after cesarean increases the odds of stillbirth compared to repeat cesarean in this population, neonatal outcomes are already known to be better under these circumstances and thus, this is not a novel finding [18–21].

This analysis is limited in lacking uterine rupture as a maternal outcome of relevance to the study question. However, the dataset includes relevant related covariates such as hysterectomy and blood transfusion as well as neonatal outcomes such as stillbirth. Other variables of interest that were unavailable but would contribute to a deeper understanding of this complex scenario include mode of delivery intention compared to actual mode of delivery. Strengths of the analysis are the large sample size, which contributes to external validity, the high quality of the data, and the breadth of antepartum, intrapartum, and postpartum variables that were included in the analysis.

## Conclusion

In conclusion, this analysis provides an evidence base that could change the paradigm of counseling in Guatemala with respect to the relationship between IPI and mode of delivery among women with a history of prior cesarean delivery. While not the only risk factor of interest in a highly complex decision-making process, this analysis provides evidence that IPI need not be a strict exclusion criterion for trial of labor after cesarean with respect to the most common maternal and perinatal/neonatal outcomes. Studying the association of this finding with trial of labor after cesarean rates would be an important area for future study.

## Data Availability

Data available on request due to privacy/ethical restrictions.

## Abbreviations

IPI: Interpregnancy interval
MNHR: Maternal and newborn health registry
